# Dipeptidyl peptidase-4 enzyme inhibition and its impacts on hepatic preneoplasia: a new avenue for liver cancer management

**DOI:** 10.3389/fphar.2025.1559303

**Published:** 2025-07-25

**Authors:** Hebatollah E. Eitah, Rabab H. Sayed, Yousreya A. Maklad, Amina A. Gamal el Din, Khaled Mahmoud, Ayman E. El-Sahar, Amani Alhejely, Amal A. Abdulbaqi, Hanan Naeim Attia

**Affiliations:** ^1^ Medicinal and Pharmaceutical Chemistry Department, (Pharmacology Group), National Research Centre, Giza, Egypt; ^2^ Pharmacology and Toxicology Department, Faculty of Pharmacy, Cairo University, Cairo, Egypt; ^3^ School of Pharmacy, Newgiza University, Giza, Egypt; ^4^ Pharmacognosy Department, National Research Centre, Giza, Egypt; ^5^ Pathology Department, National Research Centre, Giza, Egypt; ^6^ Department of Biology, Darb University College, Jazan University, Jazan, Saudi Arabia

**Keywords:** CYP2E1, CYP3A4, diethylnitrosamine, DPP-4, hepatic preneoplasia, sitagliptin

## Abstract

**Aims:**

Dipeptidyl peptidase-4 enzyme (DPP-4) was reported to be associated with immune stimulation, resistance to anti-neoplastic agents and lipid accumulation. Dysregulated DPP-4 expression was reported in various malignant tumors such as hepatocellular carcinoma. Hence, the influence of sitagliptin, an inhibitor of DPP-4 enzyme, was performed *in vitro* (HepG2 cells) and *in vivo* (mouse model of hepatic preneoplasia).

**Main methods:**

The effect of sitagliptin was investigated *in vitro via* MTT assay. The *in vivo* model of hepatic preneoplasia was conducted by weekly intraperitoneal injection of 75 mg/kg of diethylnitrosamine (DEN) for five successive weeks. Mice were treated daily with sitagliptin (50 mg/kg, p.o.) starting 1 week after DEN injection till the end of the experiment.

**Key findings:**

Sitagliptin exerted a significant cytotoxic effect on HepG2 cells, which was dependent on elevating mRNA expression of p53 and BAX/BCL2. Sitagliptin also improved serum liver enzymes and attenuated histopathological alterations in mice. These changes were accompanied by reducing liver GGT, DPP-4, CYP2E1, GGT-P, NF-κB and PCNA along with increasing CYP3A4. Furthermore, sitagliptin attenuated DEN-induced liver DNA damage and inflammation.

**Significance:**

These findings shed the light on the role of DPP-inhibitors in the future of cancer therapy and management.

## 1 Introduction

The liver is a key metabolic organ that detoxifies natural and foreign harmful chemicals, which makes it highly susceptible to carcinogenesis. Hepatocellular carcinoma (HCC) is a common type of liver cancer, which is aggressive and associated with rapid progression and poor prognosis (Memon et al., 2020). It develops via multistep processes linked to different genetic changes that eventually lead to malignant transformation of liver cells (Sarkar et al., 2020). HCC is mostly linked to extrinsic factors that directly or indirectly prompt DNA damage and chromosomal aberrations. Accumulation of uncontrolled DNA damage, results in formation of expanding foci of dysplastic hepatocytes, which progress to liver malignancy (Buitrago-Molina et al., 2013).

Treatment of HCC is complex with limited surgical resection due to risk of postoperative liver failure. Although liver transplantation is considered the best choice to increase the survival, it cannot be performed in all cases due to insufficient organs and incompatibility (Memon et al., 2020). High mortality and adverse effects associated with chemotherapy and radiotherapy highlights the need for alternative therapy in cancer management (Assar et al., 2021).

Injection of diethylnitrosamine (DEN) is used experimentally to induce HCC (Memon et al., 2020; Sarkar et al., 2020). DEN is a genotoxic compound which is bio-activated by cytochrome P4502E1 (CYP2E1) producing mutagenic DNA adducts and production of reactive oxygen species (ROS), thus promoting hepatocarcinogenesis (Kang et al., 2007; Memon et al., 2020; Sarkar et al., 2020).

Dipeptidyl peptidase 4 (DPP-4) enzyme is one of type II transmembrane glycoproteins that cleaves the X-proline or X-alanine dipeptides from the N-terminus of polypeptides such as chemokines, neuropeptides and peptide hormones (Zhao, 2019; Kawakubo et al., 2020). DPP-4 inactivates incretins including glucagon-like peptide-1 (GLP-1) and gastric inhibitory polypeptide (GIP) regulating blood glucose level (Onoyama et al., 2015; Katakami et al., 2018). DPP-4 enzyme associates with several proteins, so it plays a crucial role in cancer biology and may be considered as a useful marker for different tumors (Havre et al., 2008). In addition, DPP-4 enzyme was reported to be involved in tumor metastasis and invasion. Moreover, DDP-4 may act as either a tumor suppressor or promoter via its role with chemokines and cytokines (Almagthali et al., 2019). Previous data refer to the role of DPP-4 in the development of various chronic liver diseases like non-alcoholic fatty liver disease, hepatitis C virus infection and hepatocellular carcinoma. DPP-4 is also present in hepatic stem cells and plays a key role in hepatic regeneration (Itou et al., 2013). Studies on DPP-4 enzyme and its role in liver cancer development still did not provide a conclusive answer regarding its role in HCC (Yu et al., 2020; Busek et al., 2022).

Sitagliptin is a selective inhibitor of DPP4 that is used in management of type II diabetes (Mest and Mentlein, 2005). Recently, sitagliptin has demonstrated a hepatoprotective activity in thioacetamide-induced acute liver injury in mice (El-Kashef and Serrya, 2019), rat liver steatosis (Shen et al., 2018) and mice diabetic liver disease (Wang et al., 2021). Therefore, the present study was conducted to investigate the possible effects of the sitagliptin on *in vitro* HepG2 cells and DEN-induced hepatic preneoplasia in mice.

## 2 Materials and methods

### 2.1 *In vitro* study

Human hepatocellular carcinoma cell line HepG2 cells were obtained from Karolinska Institute, Sweden with the help of Dr. Stig Linder. Minimum Essential Medium (MEM) medium 10% fetal bovine serum (Gibco, United States) was used for culturing the cells and then they were maintained in an incubator at 37°C with a humidified atmosphere of 5% CO_2_.

### 2.2 Cytotoxic effect of sitagliptin on HepG2 cells

A Laminar flow cabinet biosafety class II level (Baker, SG403INT, Sanford, ME, United States) was used for conduction of all *in vitro* procedures in a sterile area. Fetal bovine serum (10%), 1% L-glutamine and 1% antibiotic-antimycotic mixture were all added to MEM media and used for the suspension of HepG2 cells, then cells were kept at 37°C under 5% CO_2_. A 96-well plate was used for seeding the cells at a density of 10 × 10^3^ cells/well at 37°C for 24 h under 5% CO_2_ using a water-jacketed CO_2_ incubator (Sheldon, TC2323, Cornelius, OR, United States).

Sitagliptin (3.90–250 µmole, concentration) was added to wells and cultured for 48 h, then aspiration of the medium was done followed by incubation with 40 µL 3-(4,5-dimethylthiazol-2-yl)-2,5-diphenyl tetrazolium bromide (MTT) salt (2.5 μg/mL) for 4 h. After that, 200 µL of 10% sodium dodecyl sulfate (SDS) dissolved in deionized water were added to all wells to dissolve the formed crystals as well as stop the reaction and then incubated at 37°C overnight. The development of purple formazan is an indication of cell viability (Mosmann, 1983).

Wells containing cells only served as the negative control. Doxorubicin (200 µm) was used as a positive control that is reported to give 100% lethality when used under the same conditions (Thabrew et al., 2005; EL-Menshawi et al., 2010). The absorbance was read at 595 nm using microplate multi-well reader (Bio-Rad Laboratories Inc., model 3350, Hercules, California, United States) with reference wavelength set at 620 nm. The resulting color absorbance is directly proportional to viable cell number in each sample. Dimethyl sulfoxide (DMSO<0.2%) was used as a vehicle for all treatments. The %viability was estimated using the following formula:

$$\left[1-\left(\text{AT}/\text{AC}\right)\right]*100,$$

where AT = absorbance of treatment and AC = absorbance of negative control.

### 2.3 mRNA expression of BCL2-associated X protein (BAX), B-cell lymphoma 2 (BCL2), and tumor protein (p53) in HepG2 cells using real-time PCR technique

To isolate total RNA from liver cancer cell lines, RNeasy Mini Kit (Qiagen, Hilden, Germany) supplemented with DNaseI (Qiagen) digestion step were used as reported by the manufacturer’s protocol. StepOne™ Real-Time PCR System was used to determine cDNA copy number of the liver cell line (Applied Biosystems, Thermo Fisher Scientific, Waltham, MA United States). The primer sequences are as follows: β-actin, F-CAT CCG CAA AGA CCT GTA CG, R-CCT GCT TGC TGA TCC ACA TC, accession no, DQ407611.1, BAX, F-GAT GAC CCT CTG ACC CTA GC, R-CGG GCA TTA AAG AGC TGG AC, accession no, NM_001291430.2; BCL2, F-CAA GTG TTC CGC GTG ATT GA, R-CAG AGG AAA AGC AAC GGG G, accession no, KY098799.1 and p53, F-TGG CCA TCT ACA AGC AGT CA, R-GGT ACA GTC AGA GCC AAC CT, accession no, AB082923.1. The 2^*−ΔΔCT*^ method was used for the relative quantification of the target to the reference (Ramadan et al., 2019).

β-actin was selected as the housekeeping gene due to its consistent expression under our experimental conditions, as previously validated in HepG2 cells treated with pharmacologic agents (Ramadan et al., 2019).

Housekeeping genes are favored for two reasons: they encode proteins that undergo stabilizing selection to maintain metabolic function, and they exhibit sufficient variability to detect a range of variants in the isolate collection. The nucleotide substitutions in housekeeping genes accumulate relatively slowly and remain constant enough for the method to be ideal for global epidemiology. Those housekeeping proteins are used as internal controls with presumed stability and no changes in physiological condition (Li and Shen, 2013; Muñoz et al., 2014).

### 2.4 *In vivo* study

#### 2.4.1 Animals

A total of forty male albino mice weighing 20–25 g were obtained from the animal breeding unit at the National Research Centre (Giza, Egypt). Mice were housed in laboratory standardized conditions of proper ventilation, relative humidity (55% ± 5%) room temperature (23°C ± 2°C), and a 12-h light/dark cycle. Animals were provided free access to standard chow diet and water. Approval for this investigation was granted by the Ethics Committee for Animal Experimentation at the Faculty of Pharmacy, Cairo University (Permit Number: PT 2198) and the Ethics Committee for Animal Experimentation at the National Research Centre, Giza, Egypt (18047).

#### 2.4.2 Chemicals and drugs

Diethylnitrosamine and sitagliptin were purchased from Sigma Aldrich (St. Louis, MO, United States) and Merck Sharp and Dohme Ltd. (Hertfordshire, United Kingdom), respectively.

#### 2.4.3 Experimental design

**FIGURE 1:**
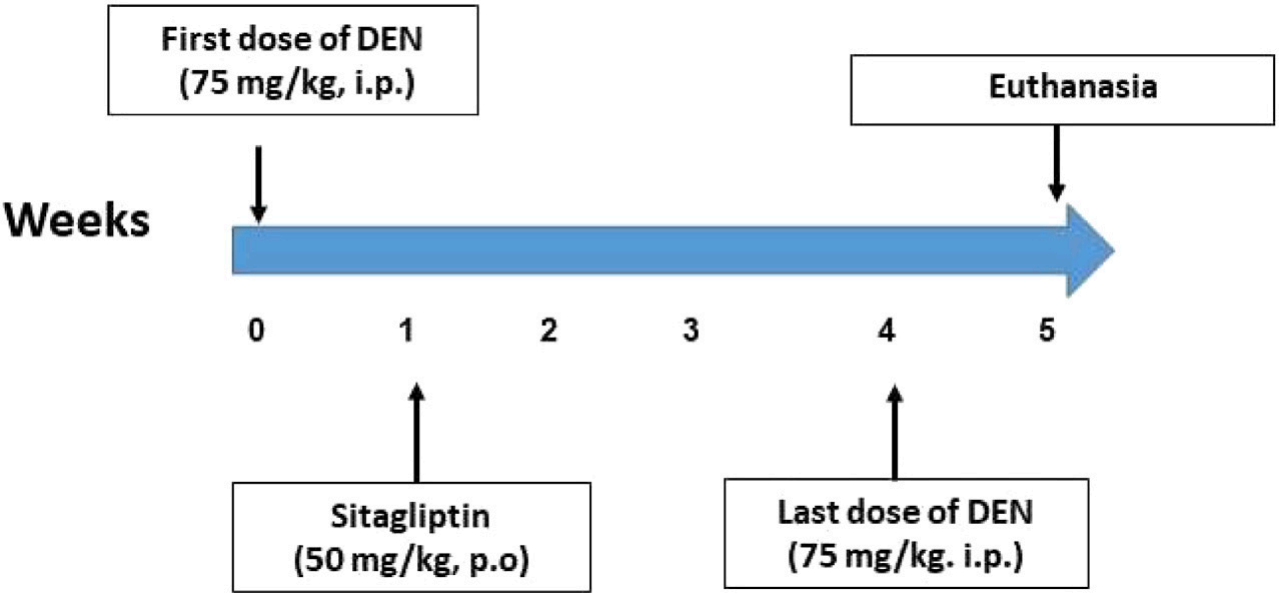
Timeline of the experiment.

Animals were randomly assigned into four groups of 10 mice each (Figure 1). Group 1 (Control): mice received saline intraperitoneally (i.p.). Group 2 (Sita): mice received sitagliptin (50 mg/kg, p.o.) dissolved in distilled water (Gault et al., 2015) for 4 weeks. Group 3 (DEN): Mice were given five weekly intraperitoneal injections of 75 mg/kg DEN dissolved in saline (Eitah et al., 2023). Group 4 (DEN + Sita): mice received DEN as in group 3, combined with daily oral sitagliptin (50 mg/kg) 1 week post-DEN injection and continued for 4 weeks.

For the calculation of the weight change percentage, the following formula was used: (Lee et al., 2012).

$$\text{Weight Change }\%=\left(\text{Final Weight}-\text{Initial Weight}\right)/\text{Initial Weight}\times 100$$

The experiment was terminated after the fifth week post-DEN induction. Blood samples were collected under light anesthesia, and then the mice were euthanized by cervical dislocation. Liver was rapidly excised, washed with ice-cold saline, dried, and weighed to record the absolute liver weight as well as relative liver weight (liver weight per 100 g of animal weight). Tissue homogenate (10%) was prepared from the right lobe for biochemical analysis. The left lobe was isolated for histopathological investigation.

#### 2.4.4 Biochemical analysis

Commercially available colorimetric kits were used for the determination of serum levels of aspartate aminotransferase (AST) and alanine aminotransferase (ALT) obtained from Teco Diagnostic (Anaheim, United States) and alkaline phosphatase (ALP) obtained from BioAssay Systems (Hayward, United States). However, mouse-specific ELISA kits were purchased from LifeSpan BioSciences, Inc. (North America) to determine the tissue level of gamma glutamyl transferase (GGT), DPP-4, tumor necrosis factor alpha (TNF-α), CYP2E1, and CYP3A4. ELISA kits for tissue interleukin (IL)-6 were provided from Mybiosource (San Diego, CA, United States). Procedural steps were strictly conducted as per the manual provided by the manufacturer.

#### 2.4.5 Comet assay

The modification of the method done by Blasiak et al. (2004) was used to perform the comet assay. Low melting-point agarose (1:10 v/v) was mixed with liver cells of each treatment group, and then pipetted to pre-coated slides with normal-melting-point agarose. In a dark environment, the slides were kept flat for 30 min at 4°C. Low melting point agarose (third layer) was then pipetted on slides and left to solidify at 4°C for 30 min. A pre-chilled lysis solution was used to keep the slides at 4°C for 60 min. Freshly prepared alkaline unwinding solution was used for immersion of the slides for 60 min away from light at room temperature. An electrophoresis run at 0.8 V/cm and 300 m Amps was applied to slides for 30 min at 4°C. Neutralizing solution was used for rinsing the slides, and then the slides were immersed in 70% ethanol and then air-dried. Slides were stained using ethidium bromide, then a Zeiss epifluorescence microscope (510–560 nm, barrier filter 590 nm) was used for visualization at 400x magnification. According to Augustyniak et al. (2016), 400 cells per group were scored. Random selection of cells that are non-overlapping was done. Subsequently, cells were visually assigned a score on an arbitrary scale of 0–3; where class 0 = no damage or tail, class 1 = tail length < nuclear diameter, class 2 = tail length ranging from 1 and 2x nuclear diameter, class 3 = tail length 2x larger than nuclear diameter (Fairbairn et al., 1995).

#### 2.4.6 Liver histopathology

Liver tissues were flushed and fixed for 48 h in 10% neutral buffered formalin. Samples were prepared to be embedded in paraffin, then cut into 5 µm thick serial sections using the rotatory microtome and mounted on glass slides for staining with hematoxylin and eosin (H&E). Sections were examined using an Olympus CX41 research microscope. Sample fixation, staining and all standard procedures were done according to Culling (2013). A CCD digital camera (Olympus SC100) which is attached to the microscope was used for the slide tissue microphotography. Digital photomicrography for the hepatic tissue sections was done at different power magnifications.

#### 2.4.7 Proliferating cell nuclear antigen (PCNA), placental glutathione S-transferase (GST-P), and nuclear factor kappa B (NF-κB) immunostaining

Immunohistochemical staining of paraffin-embedded tissue sections (5-μm) was performed as per the manual instructions. H_2_O_2_ (3%) was used for 20 min for the treatment of the deparaffinized retrieved tissue sections. This was followed by incubation with anti-GST3/GST pi antibody (Cat no. ab153949, Abcam, Cambridge, United Kingdom), anti-PCNA (Cat no. MA5-11358, Thermo Fisher Scientific Inc., Rockford, IL, United States) and anti-p-NF-κB p65 antibody (Cat no. GTX54672, GeneTex Inc., Hsinchu, Taiwan) overnight at 4°C. After washing, incubation with secondary antibody HRP Envision kit (DAKO) was done for 20 min. Then, washing with PBS was followed by incubation with diaminobenzidine for 10 min. Counterstaining with hematoxylin was done after washing with PBS, then dehydrating and clearing in xylene was done, and finally cover-slipping for microscopic examination. Leica Microsystems (GmbH, Germany) was used for analysis of data *via* a high-definition microscopic imaging system. Brownish cytoplasmic staining represents GST-P + immune reaction, while brownish nuclear staining represents PCNA + immune reaction. Regarding NF-κB, tissue images are considered positive immunoreactive by brownish nuclear and cytoplasmic staining. For quantitative measurement of area percentage for GST-P, PCNA, and NF-κB, six non-overlapping fields were randomly selected and scanned per tissue section of each sample in the immunostained sections.

#### 2.4.8 Morphometric analysis

A Leica DM-LB microscope with a JVC color video camera connected to a computer system was utilized as part of an image analysis system (Leica Qwin 500 Image analyzer, LEICA Imaging Systems Ltd., Cambridge, United Kingdom) for morphometric measurement at a magnification of ×100 on standard hematoxylin. Once the light source was adjusted, the slides to be inspected were put on the microscope. Additionally, the lighting was successfully evaluated and modified. A green mask, known as a binary image, automatically covered a lined tube that encircled the chosen regions of each parameter. The attributes of these binary images were automatically rendered in a table that included the total, mean, standard deviation, standard error, and the minimum and maximum areas measured in micrometers. Twenty fields on each slide were used for area measurement, with subsequent mean and standard error determination for all parameters examined.

### 2.5 Statistical analysis

For *in vitro* investigation, statistical analysis was carried out with the SPSS 11 program using regression analysis to compare samples with the negative control. *In vivo* data were compared using one-way ANOVA and a Tukey’s *post hoc* test with GraphPad Prism software version 6 (San Diego, CA, United States). Values of p < 0.05 were regarded as significant.

## 3 Results

### 3.1 *In vitro* experiment

#### 3.1.1 Cytotoxic effect of sitagliptin on HepG2 cells

Sitagliptin exhibited a cytotoxic effect on HepG2 cells in a dose dependent manner, with IC50: 89 µm, while at 3.9 µm sitagliptin did not exert any cytotoxic effect (Table 1).

**TABLE 1:** Cytotoxic effect of sitagliptin on HepG2 cells.

Treatment	Concentration (µmole)	Cytotoxicity (%)
Sitagliptin	250	100
	125	70 ± 1.86
	62.5	35 ± 3.68
	31.25	22 ± 3.38
	15.62	12 ± 0.81
	7.8	7 ± 0.90
	3.9	0

#### 3.1.2 Effect of sitagliptin on the mRNA expression of BAX, BCL2 and p53 in *HepG2* cells

**FIGURE 2:**
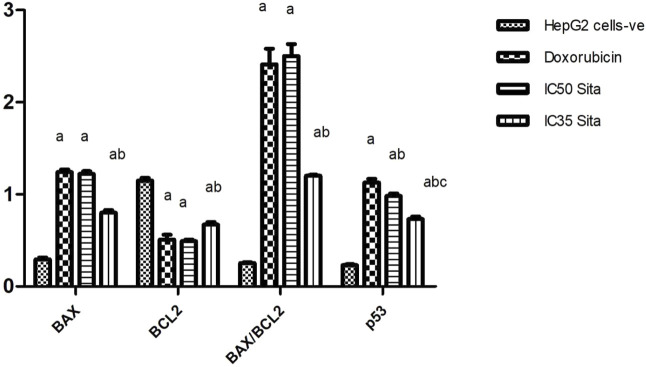
Effect of sitagliptin on the mRNA expression of BAX, BCL2, BAX/BCL2 ratio, and p53 in *HepG2* cells. ^a^ sig. from negative control HepG2 cells value, ^b^ sig. from doxorubicin, ^c^ sig. from IC 50 sitagliptin at P < 0.01. Each value represents mean ± standard error of mean (S.E.M.) (n = 4). BAX, BCL2-associated X protein; BCL2, B-cell lymphoma 2 (BCL2), P53, tumor protein.

Figure 2 illustrates a significant reduction in BCL2 expression level was reported upon treatment of HepG2 cells with the standard anticancer drug doxorubicin in a concentration of 200 µmole or with sitagliptin (IC50: 89 μM; or IC35; 62.5 µM) to reach 44%, 43%, and 58%, respectively, as compared to the negative control, untreated cancer cells. However, a significant upregulation in BAX expression level was observed in hepatic cancer cell lines treated with doxorubicin (200 µm) or sitagliptin (IC50: 89 μM; or IC35; 62.5 µM) to reach 4.23, 4.15, and 2.72 folds, respectively, as compared to the untreated HepG2 cells. Moreover, a significant increase in p53 expression level was observed upon treatment with doxorubicin 200 µmole or sitagliptin (IC50: 89 μM; or IC35; 62.5 µM) to reach 4.98, 4.31, and 3.22 folds, respectively, as compared to the untreated HepG2 cells. Similarly, a significant elevation in BAX/BCL2 ratio to reach 9.43, 9.76, and 4.69 folds, respectively, was observed in hepatic cancer cell lines treated with doxorubicin 200 µm or sitagliptin (IC50: 89 μM; or IC35; 62.5 µM) as compared to the negative control, untreated cancer cell lines.

### 3.2 *In vivo* experiment

Noteworthy, animals given sitagliptin alone did not exhibit any significant changes in all measured parameters.

#### 3.2.1 Effect of sitagliptin on final body weight in male mice expressing hepatic preneoplastic lesions induced by DEN

**FIGURE 3:**
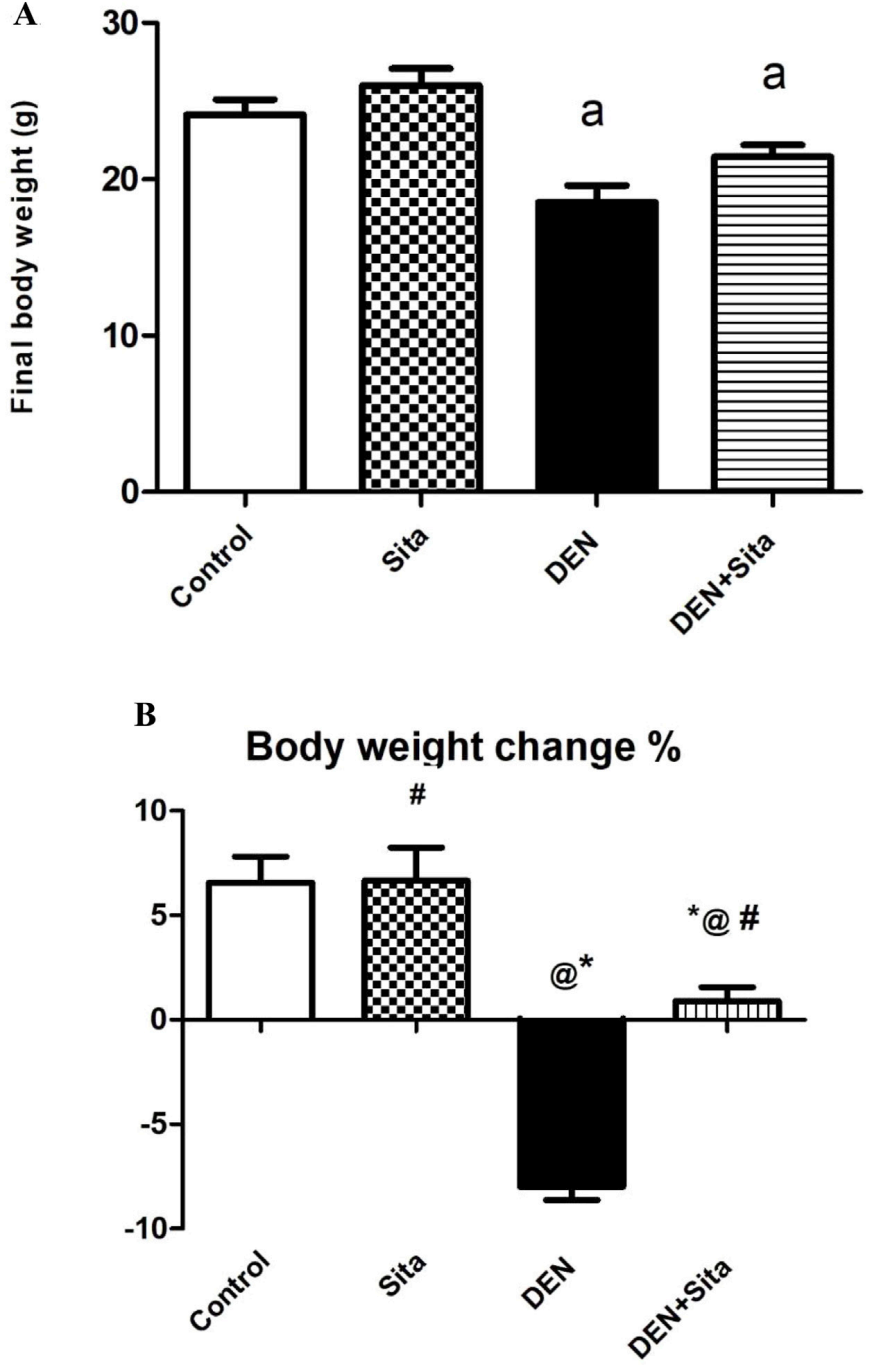
**(A)** Effect of sitagliptin on final body weight in male mice expressing hepatic preneoplastic lesions induced by DEN. ^a^ sig. from control at p < 0.05. Each value represents mean ± standard error of mean (S.E.M.) (n = 10). **(B)** Effect of sitagliptin on body weight change % in male mice expressing hepatic preneoplastic lesions induced by DEN. ^*^ sig. from control, ^@^ sig. from Sita, ^#^ sig. from DEN, at p < 0.05. Each value represents mean ± standard error of mean (S.E.M.) (n = 10).

Repeated injections of DEN to mice significantly depressed the final body weight to reach 53% as compared with the control value. Treatment with sitagliptin (50 mg/kg, p.o.) did not produce any significant change in the final body weight as compared to DEN-treated animals (Figure 3A). DEN injection resulted in a significant decrease in the body weight change % to reach −8.0 ± 0.60, while the administration of sitagliptin resulted in a significant slight rise in the body weight change % to be 0.90 ± 0.70, (Figure 3B).

#### 3.2.2 Effect of sitagliptin on absolute and relative liver weights in male mice expressing hepatic preneoplastic lesions induced by DEN

**FIGURE 4:**
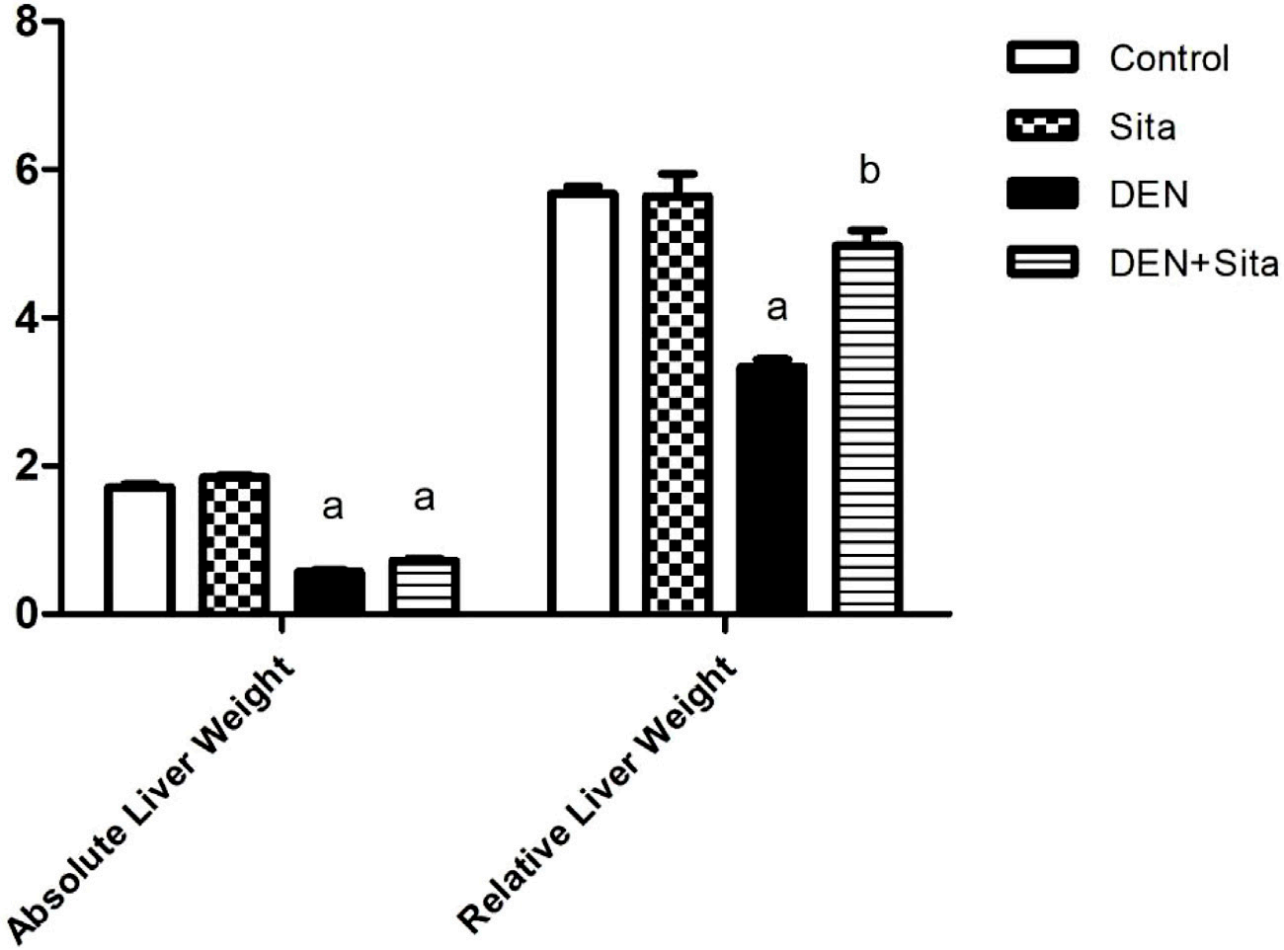
Effect of sitagliptin on absolute and relative liver weights in male mice expressing hepatic preneoplastic lesions induced by DEN. ^a^ sig. from control. ^b^ sig from DEN at p < 0.05. Each value represents mean ± standard error of mean (S.E.M.) (n = 10).

DEN-injected animals exhibited a marked decrease in absolute and relative liver weights to reach 33% and 59% as compared to the control group values, respectively. However, treatment with sitagliptin only succeeded to normalize the relative liver weight (Figure 4).

#### 3.2.3 Effect of sitagliptin on serum liver enzymes in male mice expressing hepatic preneoplastic lesions induced by DEN

**FIGURE 5:**
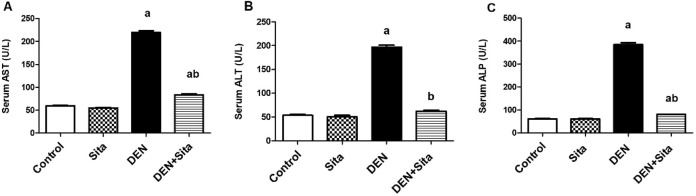
Effect of sitagliptin on serum AST **(A)**, ALT **(B)**, and ALP **(C)** in male mice expressing hepatic preneoplastic lesions induced by DEN. ^a^ sig. from control. ^b^ sig. from DEN at p < 0.05. Each value represents mean ± standard error of mean (S.E.M.) (n = 10). AST (Aspartate transaminase); ALT (Alanine transaminase); ALP (Alkaline phosphatase).

Figure 5 shows a prominent rise in serum AST, ALT and ALP activities in DEN group to reach 3.70, 3.67 and 6.31 folds, respectively as compared to the control group. Treatment with sitagliptin normalized serum ALT level and decreased serum levels of AST and ALP to reach 38% and 21%, respectively as compared to DEN group values.

#### 3.2.4 Effect of sitagliptin on tissue GGT, DDP-4, TNF-α and IL-6 in male mice expressing hepatic preneoplastic lesions induced by DEN

**FIGURE 6:**
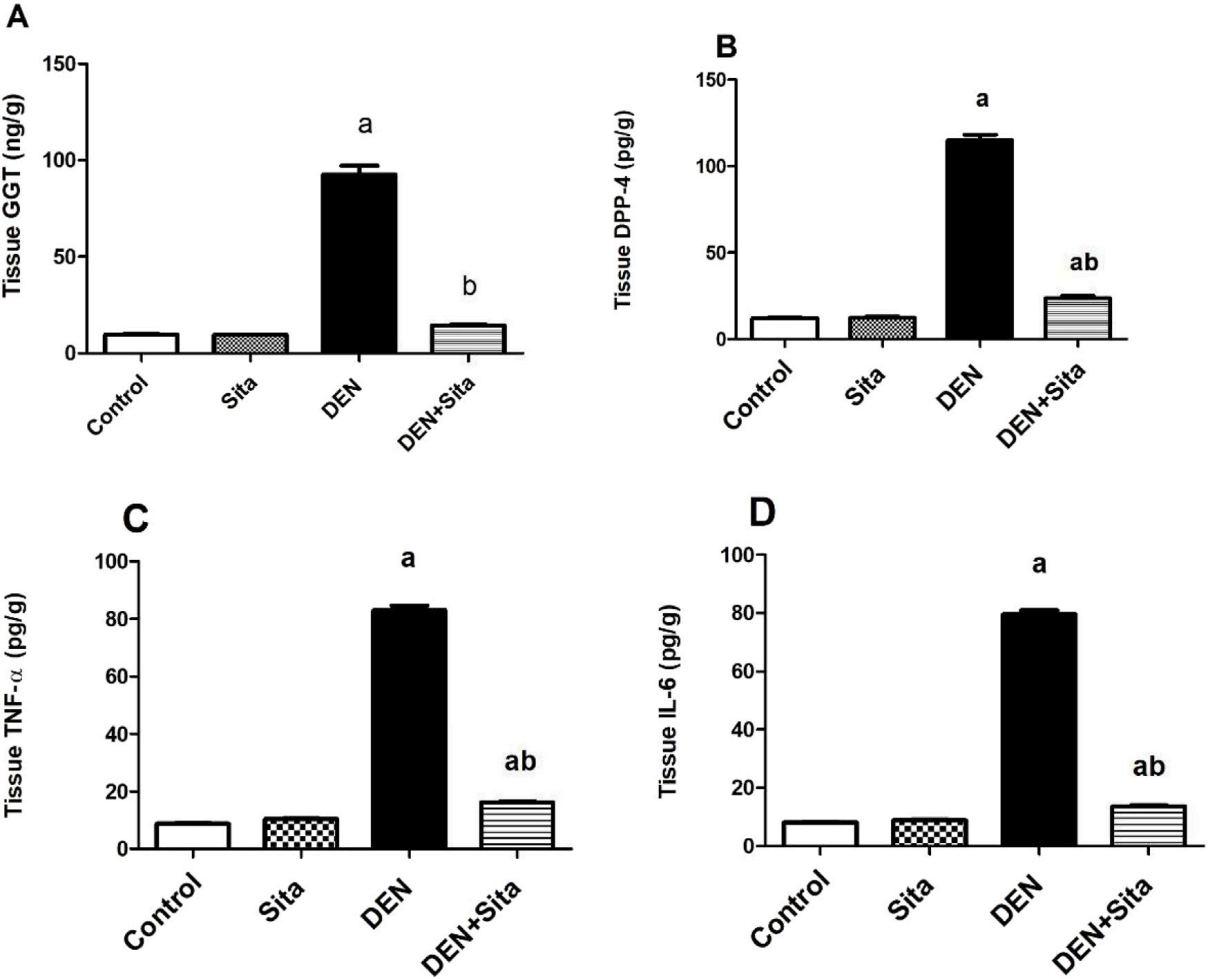
Effect of sitagliptin on tissue GGT **(A)**, DDP-4 **(B)**, TNF-α **(C)** and IL-6 **(D)** in male mice expressing hepatic preneoplastic lesions induced by DEN. ^a^ sig. from control. ^b^ sig. from DEN at p < 0.05. Each value represents mean ± standard error of mean (S.E.M.) (n = 10). GGT (Gamma glutamyl transpeptidase); DPP-4 (Dipeptidyl peptidase-4); TNF-α (Tumor necrosis factor alpha); IL-6 (Interleukin-6).

A profound rise of GGT, DPP-4, TNF-α and IL-6 tissue levels to reach 9.73, 9.40, 9.41 and 9.70 folds, respectively as compared to control group was observed in DEN-injected mice (Figure 6). In contrast, treatment with sitagliptin restored tissue GGT to normal value and significantly decreased tissue levels of DPP-4, TNF-α and IL-6 to reach 21%, 20% and 17%, respectively as compared to DEN group.

#### 3.2.5 Effect of sitagliptin on liver CYP2E1 and CYP3A4 levels in male mice expressing hepatic preneoplastic lesions induced by DEN

**FIGURE 7:**
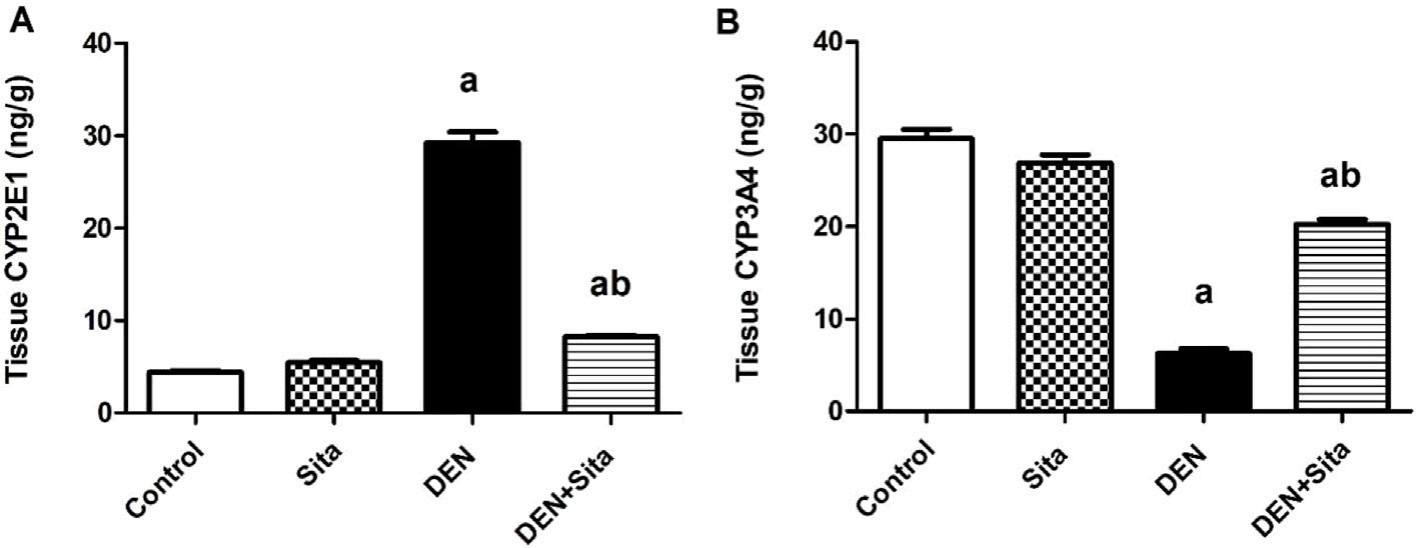
Effect of sitagliptin on liver CYP2E1 **(A)** and CYP3A4 **(B)** levels in male mice expressing hepatic preneoplastic lesions induced by DEN. ^a^ sig. from control. ^b^ sig. from DEN at p < 0.05. Each value represents mean ± standard error of mean (S.E.M.) (n = 10). CYP2E1 (Cytochrome P 2E1); CYP3A4 (Cytochrome P 3A4).

Figure 7 shows that DEN induced a significant increment in tissue CYP2E1 level to reach 6.65 folds along with a significant decline in tissue CYP3A4 level to reach 21% as compared to control group. However, sitagliptin treatment attenuated these changes as evidenced by decreased CYP2E1 level to 28% and increased CYP3A4 level to 3.23 folds as compared to DEN-injected animals.

#### 3.2.6 Effect of sitagliptin on DNA damage in male mice expressing hepatic preneoplastic lesions induced by DEN

**FIGURE 8:**
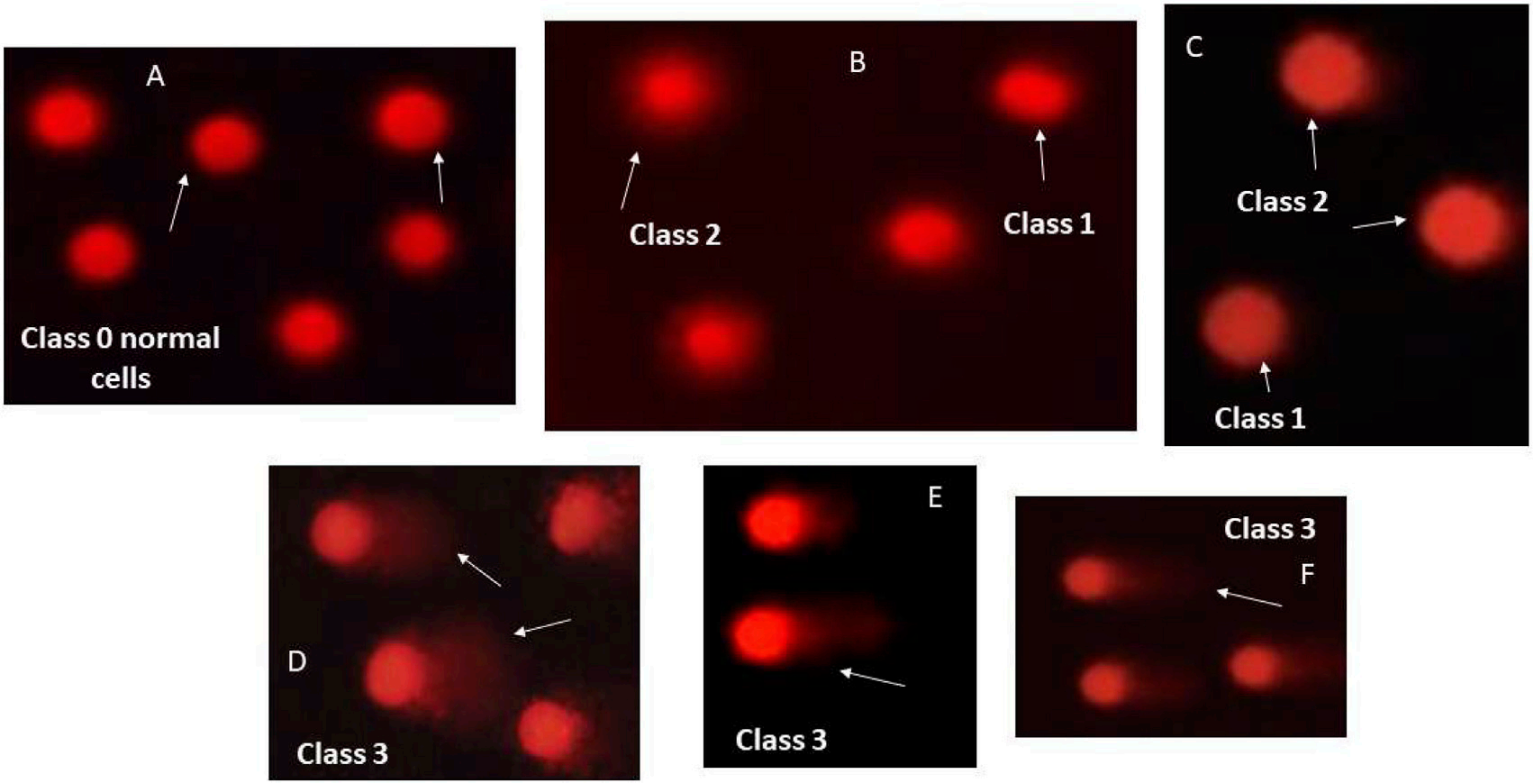
Visual scores of DNA damage using comet assay. Representative images of comet assay illustrating different types of cell classes. **(A)** class 0 normal cells without tail; **(B,C)** class 1 and 2 tailed cells; and **(D–F)**: class 3 cells with extremely tailed cells.

Data in Table 2 and Figure 8 reveal that liver tissues from DEN group exhibited marked DNA damage to reach 3.3 folds as compared to the control group. Sitagliptin administration to DEN-injected mice significantly ameliorated this DNA damage to reach 63% that of DEN group value.

**TABLE 2:** Effect of sitagliptin on DNA damage in male mice expressing hepatic preneoplastic lesions induced by DEN.

Treatment	No. of cells		Class of comet				DNA damaged cells (mean ± S.E.M)
	Analyzed	Total comets	0	1	2	3	
Control	400	30	370	25	5	0	8.00 ± 0.37
Sita	400	35	365	25	9	1	10.80 ± 0.58
DEN	400	100	300	31	29	40	26.38 ± 0.86^a^
DEN + Sita	400	65	335	19	25	21	16.50 ± 0.96^a^ ^,^ ^b^

^a^ sig. from control.

^b^ sig. from DEN, at p < 0.05. Each value represents mean ± standard error of mean (S.E.M.) (n = 10). Class 0 = no damage or tail, class 1 = tail length < nuclear diameter, class 2 = tail length ranging from 1 and 2x nuclear diameter, class 3 = tail length 2x larger than nuclear diameter.

#### 3.2.7 Effect of sitagliptin on histopathological and morphometric alterations in male mice expressing hepatic preneoplastic lesions induced by DEN

**FIGURE 9:**
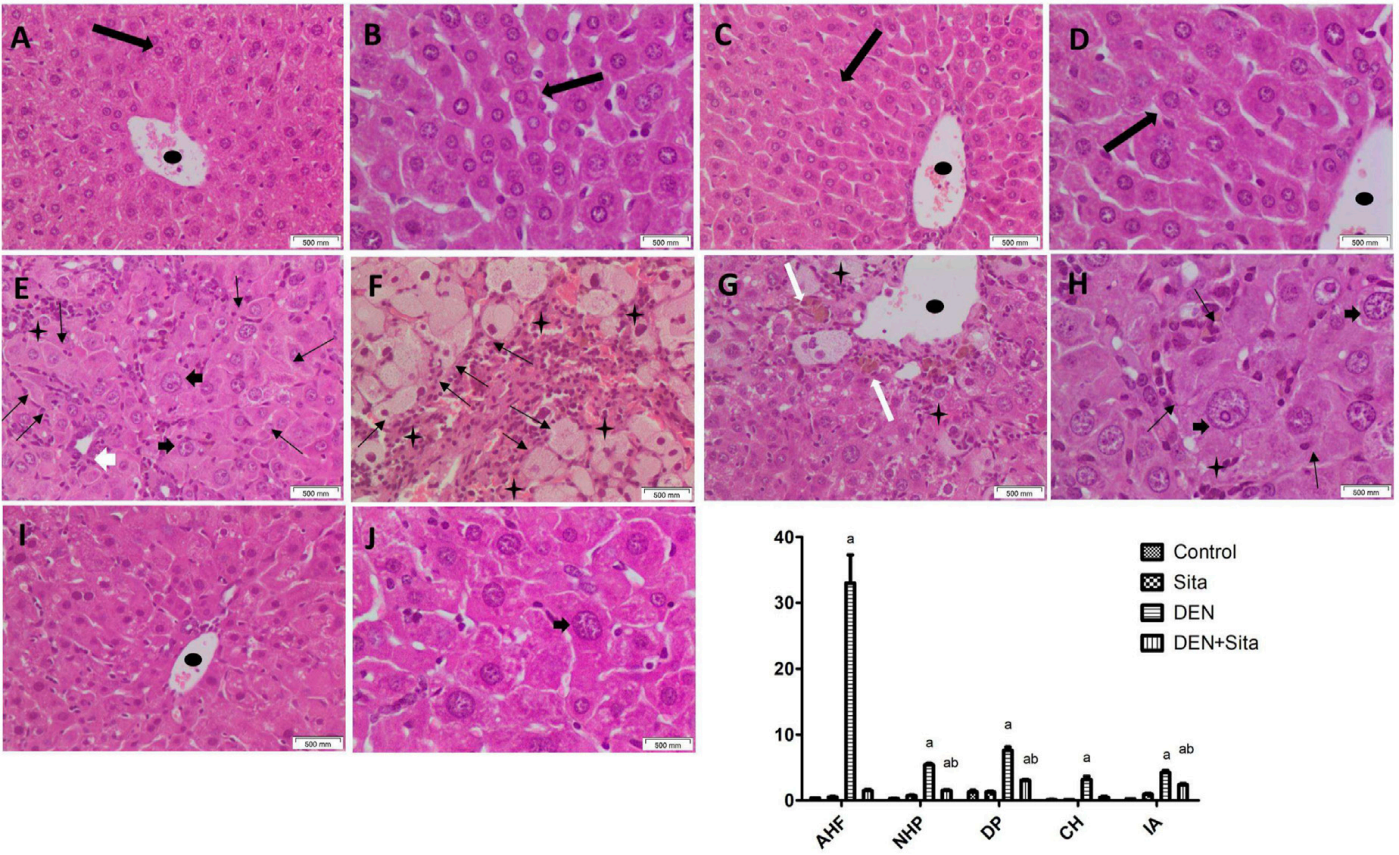
Effect of sitagliptin on histopathological and morphometric alterations in male mice expressing hepatic preneoplastic lesions induced by DEN. Photomicrographs of liver tissue sections stained with H&E. **(A,B)** (x200 and x400, respectively): Control group showing preserved architecture with hepatocytes distributed regularly in single cell thick plates radiating from a central vein. **(C,D)** (x200 and x400, respectively): Sitagliptin alone group showing normal liver architecture. **(E–G)** (x200): diseased group showing eosinophilic AHF having discrete but irregular borders, clear AHF, IA, CH and dilated central vein. **(H)** (x400): DEN-injected mice showing marked NHP, bizarre nuclei together with intra-nuclear inclusions. **(I,J)** (x200 and x400, respectively): DEN-injected mice treated with sitagliptin showing apparently normal hepatocytes with minimal residual nuclear size variation. AHF (thin black arrow), normal (thick black arrow), IA (black star), central vein (black oval shape), NHP (black arrow heads), CH (thick white arrow) and DP (white arrowhead). **(K)** Morphometric analysis (area%). ^a^ sig. from control. ^b^ sig from DEN at p < 0.05. Each value represents mean ± standard error of mean (S.E.M.) (10 sections per group). AHF (Altered Hepatocellular Foci); NHP (Nuclear Hyperchromasia and Pleomorphism); DP (Ductular Proliferation); CH (Cholestasis); IA (Inflammatory Aggregates).

Sections from control and sitagliptin alone groups revealed preserved architecture (Figures 9A–D). Hepatocytes are distributed regularly in single-cell thick plates radiating from a central vein. Sinusoids were observed between liver cell plates. However, sections from DEN group showed eosinophilic hepatocellular altered foci, bile ductular proliferation, bizarre nuclei, and inflammatory cell aggregates (Figures 9E,F). The borders of the cellular altered foci were irregular, discrete and distinguished from the surrounding tissue. The cells in the eosinophilic altered foci were large having eosinophilic cytoplasm with large nuclei in the center containing prominent nucleoli. Clear hepatocellular altered foci consisted of enlarged cells with balloon-like appearance having very pale to clear eosinophilic cytoplasm with central nuclei. Marked enlargement of the nucleus, hyperchromasia, pleomorphism (size and shape variation) and bizarre nuclei were also noticed (Figure 9F). Dilated central vein and inflammatory aggregates were observed in DEN-injected mice tissue sections (Figure 9G). Prominent nucleoli and intranuclear inclusions were also observed in DEN group sections (Figure 9H). Moreover, a significant rise in altered hepatocellular foci, nuclear hyperchromasia/pleomorphism, ductular proliferation, cholestasis, and inflammatory cell aggregates area percentages to reach 88.70, 18.57, 5.88, 21.79 and 17.38 folds, respectively was observed in DEN group as compared to control values (Figure 9K).

Sections from sitagliptin treatment group illustrated restoration of the preserved liver architecture (Figures 9I,J). The hepatocytes are regularly distributed in single-cell thick plates radiating from the central vein. Neither hepatocellular altered foci nor bizarre nuclei were observed, only minimal residual nuclear size variation was noticed. A marked decline in area percentages of nuclear ductular proliferation, hyperchromasia/pleomorphism, and inflammatory aggregates to reach 40%, 28% and 48%, respectively was also observed in liver tissue sections of mice treated with sitagliptin compared to DEN group (Figure 9K).

#### 3.2.8 Effect of sitagliptin effect on GST-P, PCNA and NF-κB immunoreactivity in male mice expressing hepatic preneoplastic lesions induced by DEN

**FIGURE 10:**
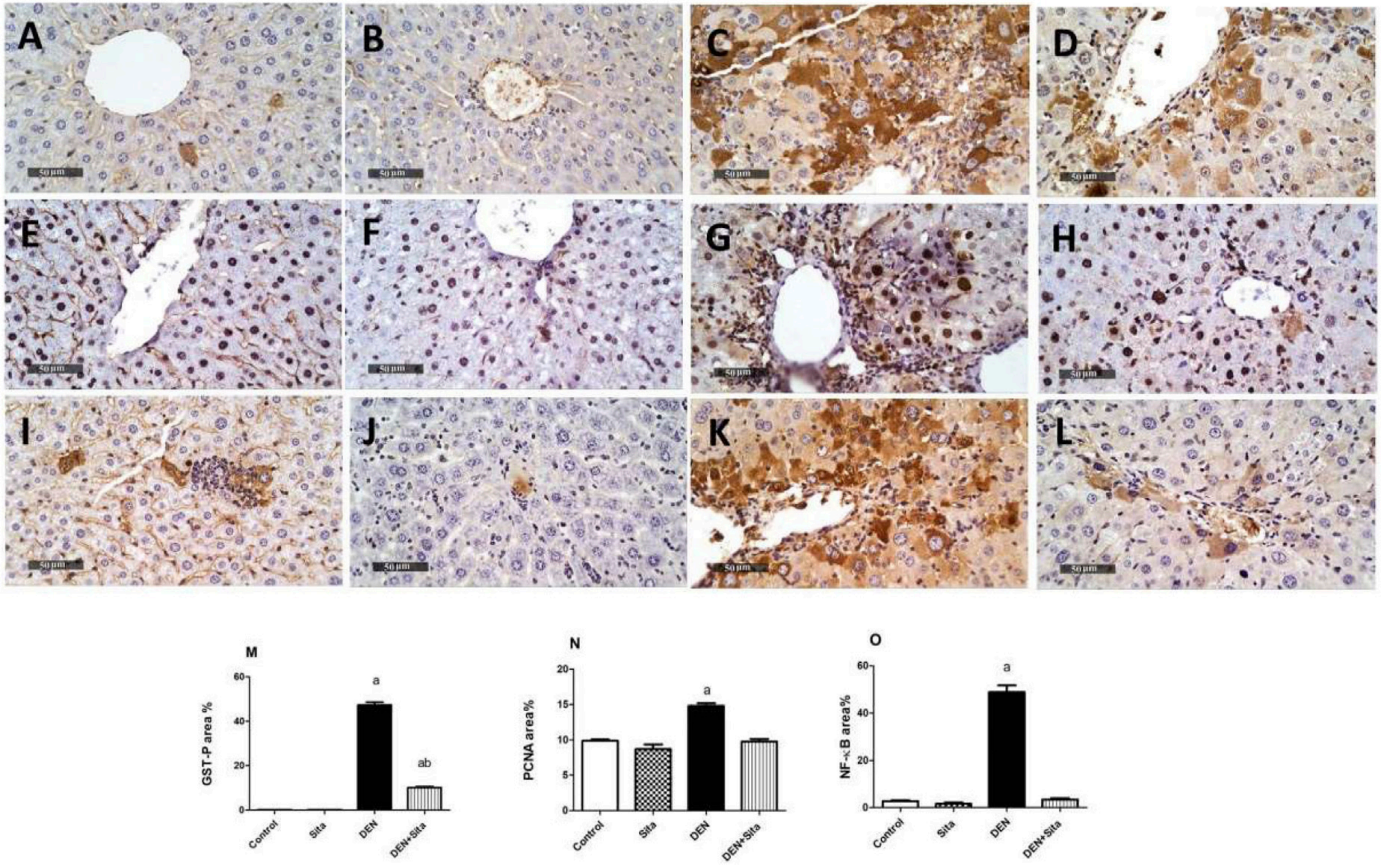
Effect of sitagliptin on GST-P, PCNA, and NF-κB immunoreactivity in male mice expressing hepatic preneoplastic lesions induced by DEN. Mice liver tissue sections A-D stained for GST-P. **(A)** Control, **(B)** Sita, **(C)** DEN, and **(D)** DEN + Sita. Liver tissue sections **(E–H)** stained for PCNA. **(E)** Control, **(F)** Sita, **(G)** DEN, and **(H)** DEN + Sita. Liver tissue sections **(I–L)** stained for NF-κB. **(I)** Control, **(J)** Sita, **(K)** DEN, and **(L)** DEN + Sita. **(M–O)** the area% of GST-P: Glutathione S transferase, PCNA: Proliferating cellular nuclear antigen, and NF-κB: Nuclear factor Kappa B, respectively. ^a^ sig. from control. ^b^ sig from DEN at p < 0.05. Each value represents the mean ± standard error of mean (S.E.M.) (10 sections per group).

As presented in Figures 10A,B, liver sections from control and sitagliptin alone mice showed homogenous GST-P distribution with few GST-P-positive cells. However, DEN-injected mice exhibited a massive positive cytoplasmic immunoreactivity for GST-P to 406 folds as compared to the control group (Figure 10C). Sitagliptin treatment attenuated these effects as indicated by lesser GST-P immunoreactivity to 21% as compared to DEN group (Figure 10D).

Similarly, liver sections from control and sitagliptin alone mice showed few PCNA-positive cells (Figures 10E,F). In contrast, DEN caused a noticeable increase in PCNA-positive cells to 1.5 folds compared with the control groups (Figure 10G). The sitagliptin treatment group manifested a clear decrease in PCNA-positive cells to 65.97% compared with the DEN group (Figure 10H).

Liver sections from control and sitagliptin alone mice demonstrated negative immunohistochemical reaction for NF-κB (Figures 10I,J). However, NF-κB was upregulated in DEN-treated animals as evidenced by positive immune reaction (18 fold) as compared to the control group (Figure 10K). Sections from sitagliptin-treated mice revealed a weaker positive expression of 7% as compared to the DEN group (Figure 10L).

## 4 Discussion

HCC, a leading cause of cancer-related mortality, typically arises in the background of chronic liver disease such as hepatitis or cirrhosis. Due to its asymptomatic progression and late-stage diagnosis, current therapeutic strategies are often inadequate, necessitating the exploration of safer and more effective chemopreventive agents (Li et al., 2021; You et al., 2021).

Results of the present study revealed that treatment of HepG2 cells with sitagliptin induced a dose-dependent cytotoxic effect with an IC50 of 89 µmole. In addition, sitagliptin treatment also resulted in a marked downregulation of the mRNA expression of the anti-apoptotic BCL2 along with a significant upregulation of the expression of the pro-apoptotic BAX, the tumor suppressor p53 and BAX/BCL2 ratio in HepG2 cells. In line, a previous study reported the cytotoxic effect of sitagliptin in HepG2 cells via its anti-proliferative and apoptogenic effects (Sarkar et al., 2017). In contrast, Sliwinska et al. (2015) reported that sitagliptin did not affect HepG2 cell viability in the dose range 0.01–200 μmole.

DEN is a chemical agent that has been extensively used for the experimental induction of hepatic preneoplasia and cancer (Gupta et al., 2010; Alsahli et al., 2021). DEN activation occurs through the CYP 450 system in the liver, especially CYP2E1, and produces DNA-adducts via an alkylation mechanism (You et al., 2021). Our results confirmed that injection of DEN to mice produced a significant depression in the final body, absolute and relative liver weights, as previously reported in the literature (Li et al., 2022). However, sitagliptin treatment succeeded in normalizing relative liver weight only. The present results also confirmed that DEN injection to mice induced marked alterations in liver function as previously reported (Memon et al., 2020). Treatment with sitagliptin attenuated DEN-induced increases in serum AST, ALT, and ALP. In agreement with our findings, sitagliptin was stated to improve liver function in non-alcoholic fatty liver disease in mice (Xu et al., 2017) and hepatic ischemia reperfusion in rats (Abdel-Gaber et al., 2019).

GGT catalyzes the main step in the degradation of reduced glutathione and is considered as a marker of liver injury (Kim et al., 2018). Elevated GGT activity leads to increased free radical production (Alam et al., 2018; Jiang et al., 2018). Furthermore, elevated GGT is associated with increased cancer risk, and may be implicated in the activation of pro-oncogenes or the inactivation of tumor-suppressor genes (Moreira et al., 2015). The present results show that sitagliptin treatment decreased tissue GGT in DEN-injected mice.

DPP-4 enzyme is an essential biomarker in cancer disease biology (Kawakita et al., 2021; Busek et al., 2022). DPP-4 enzyme is responsible for the cleavage of the N-terminal dipeptides of proline or alanine-containing peptides like incretins. In addition, DPP-4 was reported to play an important role in inflammation, immune system function, and resistance to anticancer agents (Itou et al., 2013). Moreover, high expression of the DPP-4 enzyme may contribute to the progression of liver cancer (Nishina et al., 2018).

In addition, tumor size, stage, and proliferation in HCC were related to DPP-4 enzyme level (Busek et al., 2022). Herein, tissue DPP-4 enzyme was significantly reduced after treatment with sitagliptin, which is sitagliptin’s core mechanism of action.

Cytokines are pleiotropic hormones of the immune system that play a fundamental role in the initiation, maintenance, and progression of cancer (Lee and Margolin, 2011). Neoplastic cells and tumor-associated macrophages secrete numerous cytokines as well as angiogenic growth factors that promote tumor development (Khan et al., 2017; Alsahli et al., 2021). NF-κB signaling is a key arm in the inflammatory process of the tumor microenvironment, as it regulates many genes encoding for interleukins, inducible effector enzymes, and chemokines (Zhang et al., 2021). The current data reveal that sitagliptin treatment produced a prominent reduction in DEN-induced increases in tissue TNF-α, IL-6, and NF-κB p65. These results confirm that the anti-inflammatory activity of sitagliptin is mediated through suppression of NF-κB signaling, as previously reported in DEN-injected rats (Jiang et al., 2018).

Sitagliptin’s inhibition of DPP-4 may modulate inflammatory signaling pathways, including NF-κB, through indirect effects on cytokine release and immune cell recruitment (Jiang et al., 2018).

A previous study reported that treatment with sitagliptin inhibited NF-κB p53 activation (Zheng et al., 2018). Another previous study showed that anagliptin prevented LPS-induced elevations of inflammatory cytokines in macrophages, adipocytes, and the liver in mice through suppression of NF-κB transcriptional activity (Shinjo et al., 2015).

The various metabolic activities that CYP3A4 catalyzes result in the inactivation and removal of the majority of drugs (Maideen, 2019). Further, CYP2E1 plays a critical role in the activation of many carcinogenic agents, including DEN (Gao et al., 2017). The current results show that sitagliptin treatment decreased CYP2E1 and increased CYP3A4 levels in the liver of DEN-injected mice. In parallel with current results, sitagliptin was reported to increase the expression of CYP2E1 in mice fed with a methionine/choline-deficient diet (Jung et al., 2014). Sitagliptin is predominantly eliminated via urine and feces, with 79% of the dose actively secreted unchanged in urine (Gu et al., 2020). The CYP3A4 isozyme is responsible for the limited oxidative metabolism of sitagliptin (Mistry et al., 2008), which may explain the effect of sitagliptin on CYP3A4 levels observed in the present study. The increase in CYP3A4 expression observed in DEN-injected mice following sitagliptin treatment may stem from its anti-inflammatory and hepatoprotective properties, potentially mitigating cytokine-induced suppression of CYP3A4 expression.

Most drugs are inactivated and eliminated due to the various metabolic functions mediated by CYP3A4 (Maideen, 2019). Moreover, CYP2E1 is an essential enzyme in the bioactivation of numerous carcinogens such as DEN (Gao et al., 2017). The present data suggest that sitagliptin treatment reduced liver CYP2E1 and increased CYP3A4 in the livers of DEN-injected mice. The observed induction of CYP3A4 by sitagliptin could be attributed to sitagliptin’s anti-inflammatory and hepatoprotective effects, thereby inhibiting the cytokine-induced suppression of CYP3A4 that is often observed in hepatic inflammation and preneoplasia. Inflammatory cytokines, including IL-6 and TNF, have also been found to inhibit the nuclear receptors (PXR, CAR, etc.) associated with CYP3A4 gene expression. The reduction of IL-6 and TNF-α due to sitagliptin, as seen in our study, might also have restored and/or stimulated CYP3A4 expression. This is in line with previous studies showing that DPP-4 inhibitors preserved or even increased CYP3A4 levels via suppression of inflammation (Jiang et al., 2018; Kawakita et al., 2021). Moreover, because sitagliptin is eliminated primarily unchanged and only minimally via CYP3A4-mediated metabolism (Mistry et al., 2008), we would not expect it to inhibit the activity of this enzyme and perhaps even maintain its hepatic expression when exposed to toxic injury.

Our results also show that sitagliptin treatment resulted in a significant reduction of DEN-induced liver DNA damage. This finding is in line with a study demonstrating that sitagliptin reduced total body irradiation-induced DNA double-strand breaks in the hematopoietic cells in mice via decreasing the expression of γH2AX (Wang et al., 2020), which is a concrete and sensitive molecular marker for examining DNA damage (Mah et al., 2010). Furthermore, sitagliptin markedly reduced the ratio of 8-hydroxy-2' –deoxyguanosine (8-OHdG) positive cells, another marker of oxidative DNA damage found in the liver samples isolated from animals with non-alcoholic steatohepatitis and HCC (Onoyama et al., 2015; You et al., 2021).

The results of the histopathological and morphometric analysis further confirmed our findings. Sitagliptin decreased the formation of inflammatory aggregates, nuclear hyperchromasia, and pleomorphism in liver sections. In addition, sitagliptin mitigated the altered hepatocellular foci, cholestasis, and ductular proliferation.

It was documented that GST-P is highly expressed in preneoplastic lesions and HCC induced by genotoxic carcinogens (Zhu et al., 1998; Gamal-Eldeen et al., 2016). Overexpression of GST-P in response to DEN signifies the initiation of liver carcinogenesis (Assar et al., 2021). Herein, sitagliptin treatment significantly decreased the immunoexpression of GST-P in the liver of DEN-treated mice. In agreement, Okura et al. (2017) reported that the size and number of GST-P-positive preneoplastic lesions significantly decrease after sitagliptin treatment. This could be related to its inhibitory effect on hepatic vascular endothelial growth factor (VEGF) and cluster of differentiation 31 (CD31) mRNA expressions. Proliferation has a crucial role during the initiation and promotion of carcinogenesis (Tablas et al., 2018). Synthesis of PCNA, a marker of cell proliferation, is initiated in the nucleus in late G1 phase and remains during the S phase (Arboatti et al., 2018). In the present study, sitagliptin reduced PCNA liver expression in DEN-injected mice, indicating its anti-proliferative activity.

## 5 Conclusion

Our findings demonstrate that sitagliptin exerted a significant cytotoxic effect on HepG2 cells, which was dependent on elevating mRNA expression of p53 and BAX/BCL2. Sitagliptin improved serum liver enzymes and attenuated histopathological changes in DEN-induced hepatic preneoplasia in mice via inhibition of the DPP-4 enzyme. These changes were accompanied by reducing liver GGT, CYP2E1, GGT-P, NF-κB, and PCNA along with increasing CYP3A4. Furthermore, sitagliptin attenuated DEN-induced liver DNA damage and inflammation. These findings suggest a promising effect of sitagliptin in the management of hepatic cancer initiation and progression.

## Data Availability

The original contributions presented in the study are included in the article; further inquiries can be directed to the corresponding authors.
